# Mesalazine in the initial management of severely acutely malnourished children with environmental enteric dysfunction: a pilot randomized controlled trial

**DOI:** 10.1186/s12916-014-0133-2

**Published:** 2014-08-14

**Authors:** Kelsey DJ Jones, Barbara Hünten-Kirsch, Ahmed MR Laving, Caroline W Munyi, Moses Ngari, Jenifer Mikusa, Musa M Mulongo, Dennis Odera, H Samira Nassir, Molline Timbwa, Moses Owino, Greg Fegan, Simon H Murch, Peter B Sullivan, John O Warner, James A Berkley

**Affiliations:** KEMRI-Wellcome Trust Research Programme, Nairobi, Kenya; Imperial-Wellcome Centre for Global Health Research and Section of Paediatrics, Imperial College London, Norfolk Place, London, W2 1PG UK; Baraka Health Centre, German Doctors Nairobi, Nairobi, Kenya; Department of Paediatrics and Child Health, University of Nairobi, Nairobi, Kenya; Ministry of Health, Government of Kenya, Nairobi, Kenya; Centre for Clinical Vaccinology and Tropical Medicine, Nuffield Department of Clinical Medicine, University of Oxford, Oxford, UK; Warwick Medical School, University of Warwick, Warwick, UK; Department of Paediatrics, University of Oxford, Oxford, UK

**Keywords:** Barrier function, Inflammatory bowel disorders, Malnutrition, Mucosal immunity, Tropical gastroenterology

## Abstract

**Background:**

Environmental enteric dysfunction (EED) is an acquired syndrome of impaired gastrointestinal mucosal barrier function that is thought to play a key role in the pathogenesis of stunting in early life. It has been conceptualized as an adaptive response to excess environmental pathogen exposure. However, it is clinically similar to other inflammatory enteropathies, which result from both host and environmental triggers, and for which immunomodulation is a cornerstone of therapy.

**Methods:**

In this pilot double-blind randomized placebo-controlled trial, 44 children with severe acute malnutrition and evidence of EED were assigned to treatment with mesalazine or placebo for 28 days during nutritional rehabilitation. Primary outcomes were safety and acceptability of the intervention.

**Results:**

Treatment with mesalazine was safe: there was no excess of adverse events, evidence of deterioration in intestinal barrier integrity or impact on nutritional recovery. There were modest reductions in several inflammatory markers with mesalazine compared to placebo. Depression of the growth hormone – insulin-like growth factor-1 axis was evident at enrollment and associated with inflammatory activation. Increases in the former and decreases in the latter correlated with linear growth.

**Conclusions:**

Intestinal inflammation in EED is non-essential for mucosal homeostasis and is at least partly maladaptive. Further trials of gut-specific immunomodulatory therapies targeting host inflammatory activation in order to optimize the growth benefits of nutritional rehabilitation and to address stunting are warranted. Funded by The Wellcome Trust.

**Trial registration:**

Registered at Clinicaltrials.gov NCT01841099.

**Electronic supplementary material:**

The online version of this article (doi:10.1186/s12916-014-0133-2) contains supplementary material, which is available to authorized users.

## Background

Environmental enteric dysfunction (EED, previously called environmental or tropical enteropathy) is an acquired syndrome of reduced intestinal barrier and absorptive function that is common among children living in settings where food security, safe water and facilities for hygienic waste disposal are absent or erratic [1–3]. It reflects an epidemiologic concept that lacks formal criteria for case definition but is considered to play a central role in the pathogenesis of stunting in early life. It may be a critical obstacle underlying the strikingly poor impact of even intensive nutritional and hygiene interventions on growth faltering [4]. Undernutrition (including acute malnutrition and stunting) remains the most important risk-factor for childhood mortality, responsible for an estimated 45% of all deaths among children under five years old worldwide, alongside huge burdens of short-term infectious morbidity and long-term loss of growth and developmental potential [5–10].

EED has features of an inflammatory enteropathy: histopathological changes of small intestinal villous atrophy and crypt hyperplasia are accompanied by lymphocytic infiltration of the lamina propria [11,12]. Linear growth failure is associated with detectable products of Th1 signaling and neutrophil extravasation in stool [13,14]. Systemic inflammatory activation results from breakdown in mucosal barrier function with consequent translocation of exogenous antigens alongside luminal bacteria and their products [5,15]. It has been suggested that gastrointestinal luminal contents drive this inflammation via specific enteric pathogens, a global increase in pathogen burden (due to high feco-oral transmission) or altered microbiota [4,16–18]. However, in all other inflammatory intestinal diseases of childhood, aberrant or inappropriate host response plays a critical role in the pathogenesis, even where an intraluminal trigger is evident [19]. In celiac disease, for example, although antigen-specific T cell activation is the hallmark, bystander induction of innate and other immune components contributes to tissue damage and sustained pathology [20].

A key unanswered question is whether intestinal inflammation in EED is an appropriate, adaptive response to excessive pathogen exposure that mitigates the impact of acute and chronic enteric infections or if it is maladaptive, with host-driven immunopathology contributing to mucosal barrier breakdown and growth failure. A maladaptive response would be a rational target for treatment with immunomodulatory or immunosuppressive agents such as the aminosalicylate mesalazine, which is commonly used to induce remission of mild to moderate pediatric ulcerative colitis and for maintenance of remission of ulcerative colitis and Crohn’s disease [21–23].

We conducted a pilot clinical trial with a primary objective of investigating the safety and acceptability of mesalazine as an adjunctive treatment for children with severe acute malnutrition (SAM). Acutely malnourished children are at very high risk of ongoing growth failure despite nutritional rehabilitation and their degree of intestinal barrier dysfunction has been correlated with outcome [24]. The trial was designed with intensive follow-up in order to detect any indication that the strategy was interfering with a beneficial, adaptive response. Secondary objectives were to collect hypothesis-generating data relating to inflammatory activation and growth.

## Methods

This was a single-center double-blind randomized placebo-controlled trial on the use of mesalazine alongside standard medical and nutritional care in severely acutely malnourished children. Randomization was balanced 1:1.

### Participants and setting

The study took place between June and November 2013 at the nutrition clinic of Baraka Health Centre, Mathare, Nairobi. The Baraka Health Centre (BHC) is run by ‘German Doctors’, a registered non-governmental organization, and provides free healthcare to children under five years old. Mathare is the second largest urban slum in Kenya, home to at least 200,000 people who mostly live in overcrowded iron-sheet housing with limited access to safe water and sanitation facilities [25].

Participants were recruited from among those self-presenting to BHC or via a program of active case-finding in the community conducted by local community health workers (CHWs). Eligible participants were children 12- to 60-months old with SAM, defined for the purposes of this study as mid-upper arm circumference (MUAC) <11.5 cm or bilateral pedal edema. They had uncomplicated SAM (that is, good appetite and no severe clinical illness) and were, therefore, eligible for outpatient management according to World Health Organization (WHO) guidelines [26]. EED was inferred on the basis of stunting (height-for-age z-score < −2 using the WHO 2006 growth standards) and chronic inflammation (erythrocyte sedimentation rate (ESR) >20 mm/hour). Children were excluded if they had any of the following: HIV-infection, tuberculosis, bloody diarrhea, biochemical evidence of renal or hepatic impairment, thrombocytopenia or severe anemia. Children were also excluded if they were already receiving treatment for SAM from another center, if they had medical difficulties precluding normal feeding (for example, severe cerebral palsy), if they had known pre-existing renal disease, asthma, hypersensitivity to salicylates or if they were on medication known to interfere with the action of the study drug. The exclusion criteria were decided on the basis of contraindications listed in the Summary of Product Characteristics, pre-existing conditions that the investigator group felt increased the risk to participants (for example, HIV infection, bloody diarrhea, other overt infection requiring hospital admission), or likely futility in the presence of other major medical problems (for example, tuberculosis, cerebral palsy) [27]. We did not consider concurrent or recent viral infection or administration of a live viral vaccine to be a contraindication to administration. Although Reye’s syndrome has historically been associated with salicylate (mainly aspirin) use in these circumstances, we were unable to find even a single report of Reye’s associated with mesalazine, and such cautions are not advised when it is used in the context of inflammatory bowel disease (IBD).

### Screening, enrollment and randomization

Children, 12- to 60-months old, with SAM and stunting were regarded as potentially eligible and were referred to the study team for screening. If clinical eligibility was confirmed and informed consent for participation was provided by the child’s parent or guardian, venous blood was taken for HIV testing, full blood count, ESR, creatinine, liver function tests, and film for malaria parasites, if required. Final screening with blood results took place the following day, after which eligible children were enrolled by assigning the next consecutive study number.

A randomization schedule was developed in STATA (version 12.0) with variable block sizes (two, four and six) using the following code: *‘ralloc blknum blksiz Rx, nsubj(44) osize(3) ntreat(2) saving(mys) table’* [28]. Allocations were assigned to study numbers by the trial statistician (GF). Sachets of mesalazine (Pentasa) granules and matched placebo were purchased from Ferring Pharmaceuticals (Saint-Prex, Switzerland) in 2 g foil sachets that were identical apart from labelling. Prior to initiation of the trial, sachets were disguised by application of opaque ‘black-out’ labels (Avery) and re-labelled (with the study number) by pharmacy staff independent of the trial team, according to the randomization schedule.

Dosing of drug and placebo was performed on the basis of weight, which required the contents of the 2 g sachets to be separated into smaller individual doses. Pharmacy technicians at BHC were trained to dispense the study drug using an electronic fine-scale balance (TX-323 L, Shimadzu). Doses were packed in foil pouches (purchased locally), heat-sealed to render them impermeable to light and air and labelled with the participant’s initials, study number and date. The study drug was dispensed weekly in order to minimize deterioration of the active product due to repackaging and account for changes in participants’ weight. Active and placebo granules were indistinguishable.

### Treatment

Participants were prescribed 30 mg/kg/day of mesalazine or placebo in three divided doses for the first seven days. Then, if tolerated (see below), the dosage was increased to 45 mg/kg/day for a further 21 days. They were followed up for a further 28 days after stopping the study drug (56 days total). Blood and stool were collected at days 7, 28 and 56. To account for excipients the dose of granules prescribed was 11 mg/kg three times daily for the first week, followed by 16.5 mg/kg three times daily. Because dispensing exactly to the milligram was not possible with a granular product, technicians dispensed in the range of the prescribed dose to prescribed dose + 5 mg.

If recognized side-effects of mesalazine occurred in the first week or the day 7 blood tests indicated deterioration in renal or hepatic function or blood dyscrasia (Grade 1 or 2 toxicity), the dosage was maintained at 30 mg/kg/day without unblinding. Such children were re-assessed after a week and their dose was escalated if or when it appeared to be safe to do so. The study drug was to be discontinued in the event of Grade 3 or 4 toxicity. Toxicity grades for biochemical indices were defined according to the US Division of Microbiology and Infectious Diseases’ Pediatric Toxicity Tables, 2007 [29]. Carers were asked to withhold the study drug if the child developed diarrhea, blood in stools or unexplained bruising, and to bring the child for assessment as soon as possible. The study drug was suspended until diarrheal episodes had resolved.

All children received nutritional rehabilitation with ready-to-use therapeutic foods (RUTF) conforming to WHO/UNICEF standards until they were nutritionally cured of SAM (MUAC >11.5 cm and no edema at two consecutive weekly visits), alongside a seven-day course of amoxicillin and deworming with mebendazole or albendazole according to Kenyan national guidelines [30].

### Outcomes

Primary outcomes were frequency of adverse events and compliance with the intervention, assessed via interview with the carer and weekly counting of full/empty sachets. Stool frequency and consistency was assessed at each study visit using a Kiswahili translation of the Bristol Stool Form Scale [see Additional file 1] [31]. Secondary outcomes were time to recovery, growth and a panel of inflammatory markers (see below).

### Laboratory methods

Blood tests designated for safety (full blood count, ESR, C-reactive protein, creatinine, alanine transaminase (ALT), aspartate aminotransferase (AST), gamma-glutamyl transpeptidase (GGT), bilirubin) and stool for microscopy were processed in a commercial good clinical laboratory practice (GCLP)-accredited laboratory in Nairobi (Pathologists Lancet Kenya) and results were provided the following day.

Plasma, serum and stool were kept on ice prior to freezing at −80°C pending batched enzyme-linked immunosorbent assay (ELISA) processing. The following were tested by ELISA following the manufacturers’ recommendations: fecal calprotectin (Bühlmann Laboratories AG (Schönenbuch, Switzerland) kits on stool following disruption by 30 seconds TissueLyser shaking (QIAGEN (Hilden, Germany), no beads)), plasma anti-endotoxin core immunoglobulin G (IgG EndoCAb, Hycult Biotech (Uden, The Netherlands)), plasma interferon-γ (Ebioscience (San Diego, California, USA)) and serum insulin-like growth factor-1 (R&D Systems (Minneapolis, Minnesota, USA)). Muliplex ELISA (Luminex (Austin, Texas, USA) MAGPIX system) was performed against the following targets in plasma: Eotaxin (chemokine (C-C motif) ligand (CCL)-11), GROα (growth regulated oncogene-α, chemokine (C-X-C motif) ligand (CXCL)-1), interferon-α, interleukin (IL)-1α, IL-1 receptor antagonist (IL-1RA), IL-7, IL-8, IL-10, IL-15, IL-17a, IL-22, IL-31, IP-10 (interferon-γ induced protein-10, CXCL10), MCP-1 (monocyte chemotactic protein-1, CCL2), MIP-1α (macrophage inflammatory protein-1α, CCL3), MIP-1β (CCL4), SDF-1 (stromal cell-derived factor-1, CXCL12) and tumor necrosis factor-β (TNFβ) (Ebioscience). Soluble CD14 (sCD14) was measured using an in-house ELISA (capture clone 55–3, detection clone 3-C39 both from BD (Franklin Lakes, New Jersey, USA), recombinant standard from Sigma-Aldrich Limited (Gillingham, United Kingdom)). Serum endotoxin was measured using HEK-Blue Endotoxin Detection Kit (Invivogen (San Diego, California, USA)): This test relies on a HEK293 cell line that has been stably transfected with Toll-like receptor 4 pathway genes and a secretory alkaline phosphatase that is transcriptionally regulated by NF-κB. Heat-inactivated sera (90°C for 30 minutes) were incubated in duplicate with cells for 24 hours in the presence of a detection reagent. Absorption at 620 nm was read against a standard curve.

### Statistical methods

Analysis was performed in STATA Version 12.0. We performed Mann–Whitney U tests or t-tests on log-transformed data between the arms at each time point. Growth in height and MUAC across the trial was calculated for individual participants in mm/day and compared using Mann–Whitney U tests. Fisher’s exact test was used to compare grouped variables. Comparison of the timing of adverse events was done using logrank. Raw ELISA data was analyzed in Graphpad Prism 6.0 prior to import into STATA and z-scores were calculated using WHO Anthro Version 3.2.2 STATA macros. Analyses were performed by intention to treat, except for blood and stool laboratory analyses, which were performed using data from all available specimens (that is, not including data missed due to withdrawal or failure to get specimens). For this pilot study, no cut-off was considered to indicate ‘statistical significance’, and *P*-values are provided throughout. Because the secondary analyses were intended to be exploratory and hypothesis-generating, *post-hoc* correction for multiple comparisons was not performed.

Sample size was set at 22 in each arm with reference to norms in Phase I and early Phase II research. No sample size calculation was performed and the study was not powered to formally address any outcomes at any given significance level.

### Study oversight

All participants enrolled in the study had individual written informed consent provided by a parent or guardian. The study was approved by the Kenya Medical Research Institute (KEMRI) Ethical Review Committee, the Imperial College, London, Ethical Review Committee, and the Kenya Pharmacy & Poisons Board prior to initiation. Imperial College, London, was the sponsor. Clinical trials monitoring was performed by staff from the Clinical Trials Facility, KEMRI-Wellcome Trust Research Programme. An independent Data Safety and Monitoring Board (DSMB) was established and an independent consultant pediatrician acted as local safety monitor. Neither the sponsor nor any other party except the named investigators had any role in the design of the study, interpretation of the results, content of manuscripts or decision to publish. The trial was registered at http://clinicaltrials.gov/ct2/show/NCT01841099.

## Results

**Figure 1:**
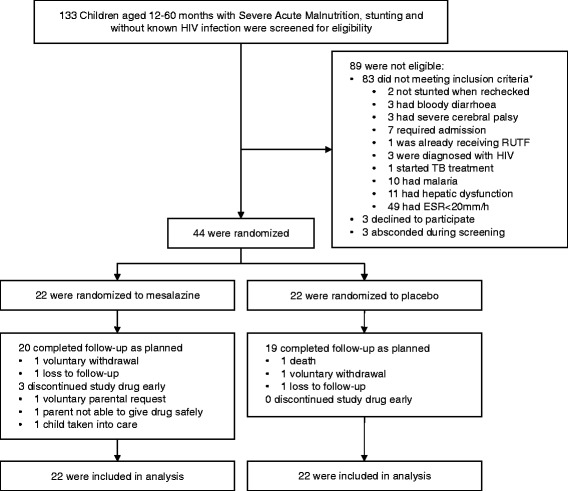
**Trial flow diagram:** *Some children had multiple reasons for being ineligible.

From June to September 2013, 133 children with SAM and stunting were screened for eligibility. Forty-four children completed screening procedures and were eligible, all of whom were enrolled (Figure 1). The arms were well balanced in terms of clinical, anthropometric and demographic characteristics at baseline (Table 1).

**Table 1:** **Baseline characteristics**

**Baseline characteristics**	**Mesalazine arm**	**Placebo arm**
Number enrolled	22	22
Age (months)	19 (14 to 32)	19 (15 to 29)
Sex: Male	10 (45)	11 (50)
Female	12 (55)	11 (50)
Household income category (USD/day)^a^	$2 to $4	$2 to $4
Number of people living in the house	5 (4 to 7)	5 (4 to 5)
Access to improved drinking water source^b^	100%	100%
Access to improved toilet facility	50%	41%
MUAC (cm)	12.9 (11.2 to 14.4)	12.7 (11.2 to 13.8)
Height-for-age z-score	−3.13 (−3.83 to -2.71)	−3.73 (−4.60 to -2.81)
Nutritional edema	15 (68)	17 (77)
Current breastfeeding^c^	11 (50)	5 (23)
Symptoms: Fever	10 (45)	8 (36)
Cough	9 (41)	6 (27)
Diarrhea	5 (23)	5 (23)
Biochemistry: Creatinine (umol/L)	18 (17 to 23)	19 (17 to 22)
AST (IU/L)	37 (27 to 52)	40 (32 to 49)
ALT (IU/L)	24 (12 to 31)	17 (15 to 30)
CRP (mg/L)	6.0 (1.3 to 10.8)	6.8 (1.6 to 42.2)
Hematology: Hemoglobin (g/dL)	10.3 (7.9 to11.2)	9.6 (8.2 to 10.6)
WBC count (x 10^9^/L)	12.0 (9.9 to 15.1)	11.8 (9.3 to 13.6)
Platelet count (x 10^9^/L)	555 (423 to 666)	536 (282 to 692)
ESR (mm/hour)	32 (24 to 38)	33 (28 to 44)

^a^Median and IQR for both arms all fell within the $2 to $4 category. ^b^Mostly communal taps from which water must be purchased. Lack of resources was reported to limit access. ^c^ *P* = 0.06. Data are medians (inter-quartile range) or numbers (%). ALT, alanine transaminase; AST, aspartate aminotransferase; CRP, C-reactive protein; ESR, erythrocyte sedimentation rate; MUAC, mid-upper arm circumference; WBC, white blood cell.

### Follow-up and compliance

Following completion of the interventional phase of the study at day 28, one child in each arm was voluntarily withdrawn from the study by their carer (in both cases because the family planned to travel away from Nairobi) and one child in each arm was lost to follow-up. Compliance with the study drug administration schedule was high in both arms (Table 2). Early discontinuation of the study drug occurred in three children, in each case for reasons unrelated to the drug itself and all three remained in follow-up: one discontinuation at day 14 was at parental request, another at day 14 was initiated by the study team because the parent was unable to give the drug as prescribed, and the third at day 22 was because the participant was taken into protective custody. All three were in the mesalazine arm.

**Table 2:** **Outcomes**

		**Mesalazine arm**	**Placebo arm**	***P***
**Safety and compliance**				
Deaths		0	1	0.32
Hospitalizations^a^		0	2	0.15
Total adverse events		35	27	0.53
Acute watery diarrhea		7	8	0.53
Bloody diarrhea		0	2	0.32
Chronic diarrhea		1	1	1.00
Gastroenteritis (with vomiting)		5	1	0.15
Giardiasis		6	6	1.00
Oral candidiasis		3	0	0.08
Upper respiratory tract infection		3	5	0.64
Lower respiratory tract infection		3	1	0.30
Tuberculosis		1	1	1.00
Malaria/non-specific febrile illness		4	3	0.43
Rash		2	2	1.00
Total gastrointestinal adverse events		23	16	0.27
Total diarrheal adverse events		8	11	0.65
Unable to dose-escalate at day 7		1 (5)	7 (32)	0.05
Compliance (%)		93 (71 to 98)	97 (82 to 99)	0.44
**Anthropometry and recovery**				
Recovery: nutritionally cured at day 56		13	12	1.00
Recovery: RUTF-free days at day 56		9 (0 to 21)	9.5 (0 to 21)	0.94
Nutritional edema	Baseline	15/22 (68)	17/22 (77)	0.72
	Day 28	7/22 (32)	6/22 (27)	1.00
	Day 56^b^	3/20 (15)	2/19 (11)	1.00
MUAC (mm)	Baseline	12.9 (11.2 to 14.4)	12.7 (11.2 to 13.8)	0.56
	Day 28	13.7 (11.8 to 14.7)	13.2 (12.2 to 14.6)	0.82
	Day 56	13.9 (12.5 to 14.8)	13.6 (13.0 to 15.1)	0.59
MUAC growth rate (mm/day)	To day 28	0.23 (0.06 to 0.37)	0.31 (0.18 to 0.61)	0.15
	To day 56	0.12 (0.08 to 0.21)	0.23 (0.14 to 0.33)	0.01
Height growth rate (mm/day) to day 56		0.31 (0.19 to 0.38)	0.32 (0.21 to 0.38)	0.94
Height-for-age z-score	Baseline	−3.13 (−3.83 to -2.71)	−3.73 (−4.60 to -2.71)	0.29
	Day 28	−3.17 (−4.04 to -2.61)	−3.70 (−4.20 to -2.73)	0.42
	Day56	−3.24 (−4.10 to -2.68)	−3.46 (−4.14 to -2.68)	0.79

^a^Includes the one child who died in hospital. ^b^Excluding children who were unable to be assessed. Data are medians (inter-quartile range) or numbers (%) unless otherwise stated. MUAC, mid-upper arm circumference; RUTF, ready-to-use therapeutic food.

### Adverse events and toxicity

Two children required hospitalization during the course of the study, both with diarrhea, dehydration and sepsis, one of whom died. Both were in the placebo arm. One child in each arm was started on tuberculosis treatment on the basis of clinical score and poor response to nutritional rehabilitation. One child in the placebo arm was newly diagnosed with sickle cell disease.

**Figure 2:**
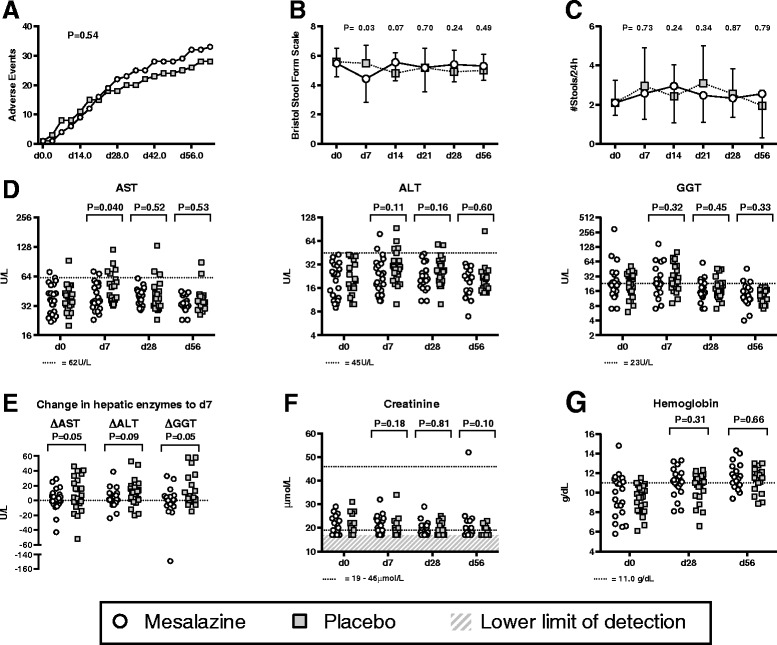
**Safety and toxicity.** Cumulative timing of adverse events between the arms **(A)**. Stool consistency (Bristol Stool Form Scale) and frequency in the 24 hours preceding clinical review **(B,C)**. Hepatic enzymes, aspartate aminotransferase (AST), alanine transaminase (ALT) and gamma-glutamyl transpeptidase (GGT), for all participants in the study **(D)**. Change in hepatic enzymes from baseline to day 7 **(E)**. Creatinine and hemoglobin for all participants **(F,G)**. Differences between arms at baseline are highlighted if *P* <0.1, upper limit of normal (alongside lower limit for creatinine) illustrated where appropriate.

Non-severe adverse events and illness episodes were common, as expected in this vulnerable patient population, but there was no suggestion of excess risk associated with mesalazine treatment either in total adverse events, adverse events involving the gastrointestinal tract, or those associated with diarrhea (Table 2 and Figure 2A). At day 7, children in the mesalazine arm had firmer stool consistency (Figure 2B, 2C).

The most common previously reported side-effects of mesalazine, apart from gastrointestinal problems, are headache and rash. Most children were pre-verbal and would have been unable to report headache but there were no concerns from carers about irritability, restlessness or problems with sleeping. Four children had an infectious rash during the course of the study but none were considered related to treatment: two had impetigo (both mesalazine arm), one had chickenpox (placebo) and one had scabies (placebo).

Dose escalation at day 7 was delayed in eight participants: seven had developed elevated transaminases compared to their enrollment results and one child was admitted to hospital on the day of their scheduled visit. Of these eight participants, seven were in the placebo arm and one was in the mesalazine arm. Transient elevation of liver enzymes compared to enrollment was common at day 7 and was more pronounced in the placebo arm (Figure 2D, 2E). No Grade 3 or 4 toxicities were encountered in the study.

Creatinine concentrations across the trial were low. A single elevated concentration occurred at day 56 in a child in the mesalazine arm who had achieved nutritional recovery (Figure 2F). The child remained well and when tested three months later the creatinine concentration was 27 μmol/L.

Thirty-four (77%) children were anemic (Hb <11.0 g/dL) at enrollment. Rates of resolution were similar in the two arms (Figure 2G).

### Growth and recovery

At completion of the 56-day follow-up period, nineteen children (34%) had not recovered, had died, or were not remaining in follow-up, nine in the mesalazine arm and ten in the placebo arm. At day 56, there were no differences in RUTF-free days or resolution of edema between the arms (Table 2). The rate of increase in MUAC was higher in the placebo than in the mesalazine arm, although this was less pronounced among those children who were enrolled without edema [see Additional file 1]. There were no differences in linear growth rate or change in height-for-age z-score (Table 2).

**Figure 3:**
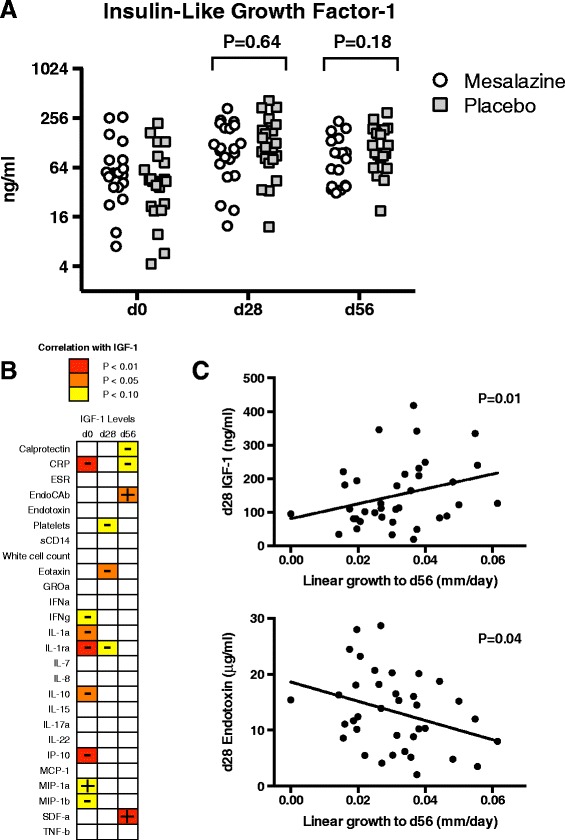
**IGF-1 and growth.** Insulin-like growth factor-1 (IGF-1) increases during follow-up **(A)**. Enrollment IGF-1 had negative correlations with several inflammatory markers (**B**, color indicates statistical significance level, + or – indicates a positive or negative correlation). This effect reduced between IGF-1 and concurrent inflammatory markers at later time points. Concentrations of IGF-1 and endotoxin correlated positively and negatively (respectively) with linear growth over the course of the study **(C)**.

Insulin-like growth factor-1 (IGF-1) increased from enrollment to day 28 in both arms, and was maintained to day 56 (Figure 3A). Enrollment IGF-1 concentration had a strong inverse correlation with a number of inflammatory mediators (for example, C-reactive protein (CRP), *P* = 0.008), which was less apparent at the later time points (Figure 3B). IGF-1 concentrations at day 28 correlated strongly with the rate of height growth both to day 28 and day 56 (Figure 3C).

### Inflammatory indices

**Figure 4:**
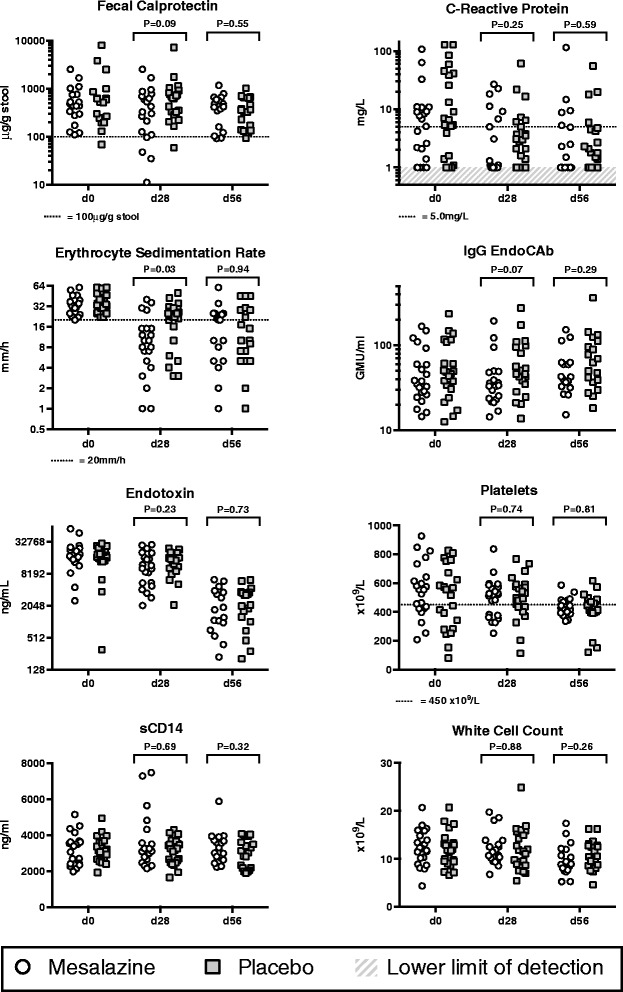
**Impact on inflammatory activation.** Key clinically important and gut-specific inflammatory markers between arms. Differences at baseline are highlighted if *P* <0.1. Upper limit of normal illustrated where appropriate.

Intestinal inflammation was evident in most children at enrollment, with 43 (97%) having fecal calprotectin higher than 100 μg/g [32]. Systemic inflammatory activation was evident, which declined during the course of the trial. Point estimates of most of the clinically important or gut-specific inflammatory markers were lower in the mesalazine arm on completion of treatment at day 28 (Figure 4). Differences between the arms were not sustained to day 56. Cytokine concentrations in plasma were similar between the arms at all time points [see Additional file 1].

## Discussion

This is the first controlled trial of a directly immunomodulatory agent in children with SAM. Despite being eligible for outpatient management with ‘uncomplicated’ SAM, intestinal and systemic inflammatory activation was very common. Baseline levels of fecal calprotectin were higher than has been reported from healthy control populations in high-income countries and sub-Saharan Africa, approaching those seen in active IBD [33,34]. Treatment with mesalazine was safe. The frequency of acute illness episodes was similar in both arms, and there was no excess gastrointestinal morbidity to suggest a disadvantageous effect on gut mucosal homeostasis. The trial was not intended to be powered to detect rates of resolution of edema, recovery of acute malnutrition or linear growth. Individual growth trajectories revealed greater MUAC growth in the placebo arm to day 56 but because edema can alter MUAC and most of the children in this study were edematous at enrollment, the clinical significance is unclear.

In pediatric IBD, linear growth impairment correlates with cytokine-mediated depression of the growth hormone – IGF-1 axis, and a similar effect was evident among participants in this trial [35–37]. It is, therefore, encouraging that the mesalazine arm achieved modest reductions in multiple clinically-relevant and gut-specific biomarkers compared to placebo after 28 days treatment. If EED represented an essential level of inflammatory activation and immune-surveillance in the face of a high pathogen burden, we would have expected the opposite result: that mesalazine would have been associated with a loss of control at the gut mucosal surface, increased levels of bacterial translocation and systemic immune activation, alongside development or worsening of gastrointestinal symptoms. Instead, we have demonstrated that pharmacologically-mediated reduction in enteric inflammation is well tolerated, thereby providing the first clear evidence that EED is likely to be at least partly maladaptive and, consequently, a potential direct target for therapy. New therapies are urgently required because trials of nutritional support, hygiene-based interventions, probiotics and empiric treatment for small intestinal bacterial overgrowth or specific pathogens have failed to show clinically significant improvements in linear growth or EED activity in children [4,38–40]. We suggest that failure to control host-driven inflammatory activation may have been a barrier to efficacy of these interventions.

An important limitation of our study is that enteric inflammation is inferred on the basis of indirect blood or fecal markers. A recent expert working group concluded on the basis of the myriad ethical and logistical challenges inherent in performing gastrointestinal endoscopy on young children, that ‘definition of environmental enteric dysfunction will need to rely on biomarkers alone, without biopsy data to connect functional and structural changes’ [1]. Importantly, the high levels of fecal calprotectin and systemic inflammatory activation demonstrated in this study are more consistent with inflammation in the colon than in the small intestine [41–43]. In view of the fact that sigmoidoscopy can be achieved far more easily than full colonoscopy and without the need for bowel preparation or deep sedation, if colonic inflammation were a major component of EED, it might be substantially more amenable to serial tissue-based monitoring in clinical trial settings. Biopsy studies of colonic architecture in children at risk of EED have not been reported to our knowledge.

The enrollment of children who were severely acutely malnourished as well as stunted was ethically appropriate because children with SAM are at the highest risk of continued growth disturbance, illness and death, meaning that they have the most to gain from any potential new intervention. However, even though these results suggest a maladaptive inflammatory enteropathy, it may be that ensuring optimal capacity to respond to a new pathogen challenge takes precedence over optimizing growth in the context of SAM, where vulnerability to major infection is intense. Targeting those with moderate acute malnutrition or non-acutely malnourished children would be likely to increase the chances of detecting any nascent IGF-1-mediated linear growth benefit and presents the most plausible scenario under which such interventions could be used in the field. In this regard, a limitation of the study is that the inflammatory enteropathy found in kwashiorkor (which was present in 73% of those enrolled) may be qualitatively different than that present in the context of other forms of acute malnutrition, and to the form of EED that appears to be prevalent with stunting. Results of the current trial should not be considered generalizable either to children with stunting but without acute malnutrition or to populations of moderately or severely malnourished children without high prevalence of kwashiorkor, and future studies in such groups will need to take a similarly cautious and thorough approach as we have here [12]. That said, such studies should consider the use of more intensive treatment schedules because although mesalazine is a good agent for maintenance of remission in IBD, it is less effective in induction. Difficulty in diagnosing tuberculosis in acutely malnourished children may preclude the use of systemic immunosuppressants, but longer courses or higher doses of mesalazine could reasonably be trialed. Furthermore, although EED occurs in all age groups, irreversible stunting mainly occurs in the first two years of life, and may even be apparent at birth [15,44]. Treatment prior to the development of clear evidence of growth failure may be optimal, but this requires the development and refinement of point-of-care biomarkers for EED. Fecal calprotectin may be a suitable marker: point-of-care tests already exist, and evaluation against dual sugar absorption tests (currently the most well-established biomarkers of EED) should be a research priority. The possibility of targeting short-term medical treatments towards this key early window of vulnerability to linear growth failure, which is also the peak period for development of acute malnutrition and major infectious diseases, means that clinical benefit for individual children might be possible even if modification of environmental determinants was minimal. This reflects social and political realities in many of the settings where EED is endemic.

Our results have highlighted that elevation of liver enzymes is reasonably common in uncomplicated SAM (8% of those screened) and that current management strategies may be associated with a transient exacerbation. Fatty infiltration of the liver is a recognized feature of kwashiorkor and could be exacerbated by a sudden switch towards a plentiful and very high-fat diet [45]. That RUTF is safe and effective for use in the community is partly a factor of its high lipid content (which restricts bacterial growth) and it is likely that any risk associated with modest increase in liver enzymes is outweighed by the proven benefits of community-based care. However, there may be a subset of patients in whom this presents a particular problem, especially as RUTF is starting to be used more commonly in the more-unwell group of hospitalized severely acutely malnourished children.

The participants involved in this trial represented a uniquely vulnerable population. Detection of a severely acutely malnourished child during community screening was frequently an indicator of multiple social, nutritional and medical problems affecting the whole family. The provision of appropriate ancillary support was an essential component of our trial methodologies, made possible because of the comprehensive integration of medical and social services and long history of community engagement in Mathare by ‘German Doctors’, which made this setting a particularly appropriate one in which to conduct the trial. Where required, parents and siblings were provided with a daily food ration, free medical care and access to social support and advice. Study visits took place at participants’ home to minimize the opportunity cost of attending the clinic and community health workers performed regular home visits in order to provide support and assess for additional needs. The same benefits were afforded to all children screened for enrollment regardless of their eligibility.

## Conclusions

In summary, we have reported the first controlled trial directly targeting the host inflammatory response in children with SAM. The trial is conceptually novel in considering EED as a maladaptive host response to environmental challenge and has provided pilot evidence of safety. Larger trials will be needed to assess efficacy but our finding of concordance of a range of inflammatory markers encourages the design of such studies.

## Additional file

Additional file 1:
**Swahili version of Bristol Stool Form Scale.** **Figure S1.** Individual mid-upper arm circumference (MUAC) trajectories for participants in the mesalazine arm (left) or placebo arm (right), grouped according to whether they were edematous (top) or non-edematous (bottom) at baseline assessment. **Figure S2.** Plasma cytokine concentrations between the arms. Differences between arms at baseline are highlighted when *P* <0.1.

## Abbreviations

ALT: alanine transaminase
AST: aspartate aminotransferase
BHC: Baraka Health Centre
CHW: community health worker
CRP: C-reactive protein
DSMB: Data, Safety and Monitoring Board
EED: environmental enteric dysfunction
ELISA: enzyme-linked immunosorbent assay
EndoCAb: anti-endotoxin core immunoglobulin G
ESR: erythrocyte sedimentation rate
GGT: gamma-glutamyl transpeptidase
Hb: hemoglobin
IBD: inflammatory bowel disease
IGF-1: insulin-like growth factor-1
IL: interleukin
MUAC: mid-upper arm circumference
RUTF: ready-to-use therapeutic food
SAM: severe acute malnutrition
sCD14: soluble CD14
WHO: World Health Organization
