# Supplementary material for: Mesalazine in the initial management of severely acutely malnourished children with environmental enteric dysfunction: a pilot randomized controlled trial
Source: BMC Med. 2014 Aug 14;12:133. doi: 10.1186/s12916-014-0133-2 (PMC4243388; doi:10.1186/s12916-014-0133-2)

**Mesalazine in the initial management of severely acutely malnourished children with environmental enteric dysfunction:**

**a pilot randomized, controlled trial**

**ADDITIONAL FILE 1 CONTENTS**

Swahili version of Bristol Stool Form Scale: Page 2

Supplementary Figures 1 and 2: Page 3 – 5

**Swahili version of Bristol Stool Form Scale:**

Developed from <http://commons.wikimedia.org/wiki/File:Bristol_stool_chart.svg>

**
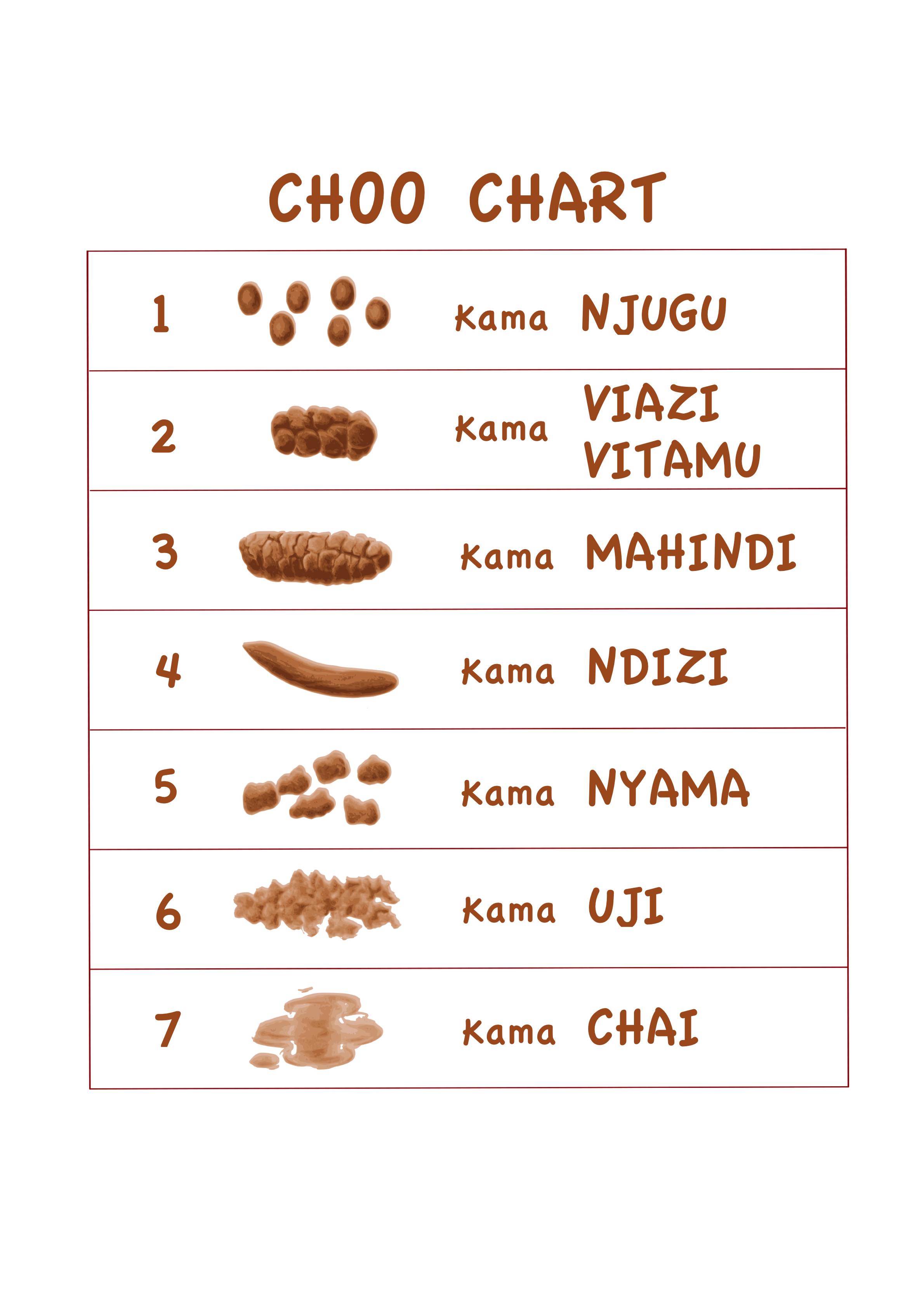
**

**Supplementary Figure 1:**


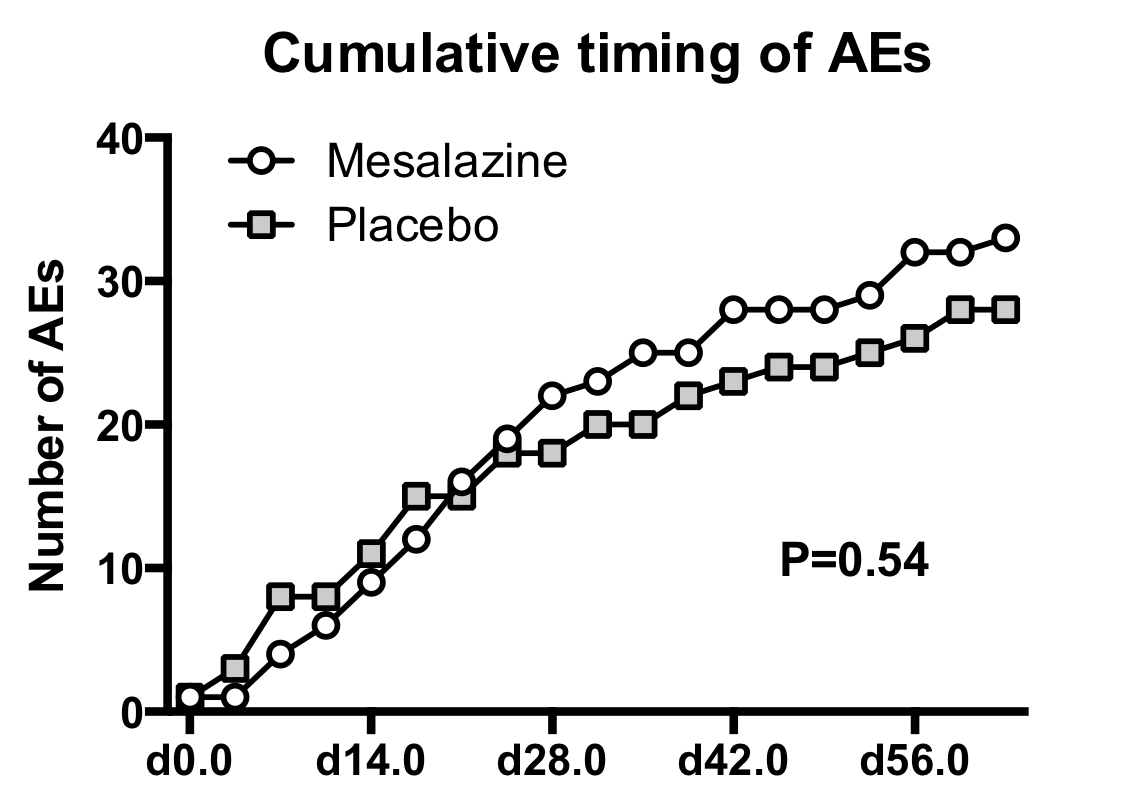


**
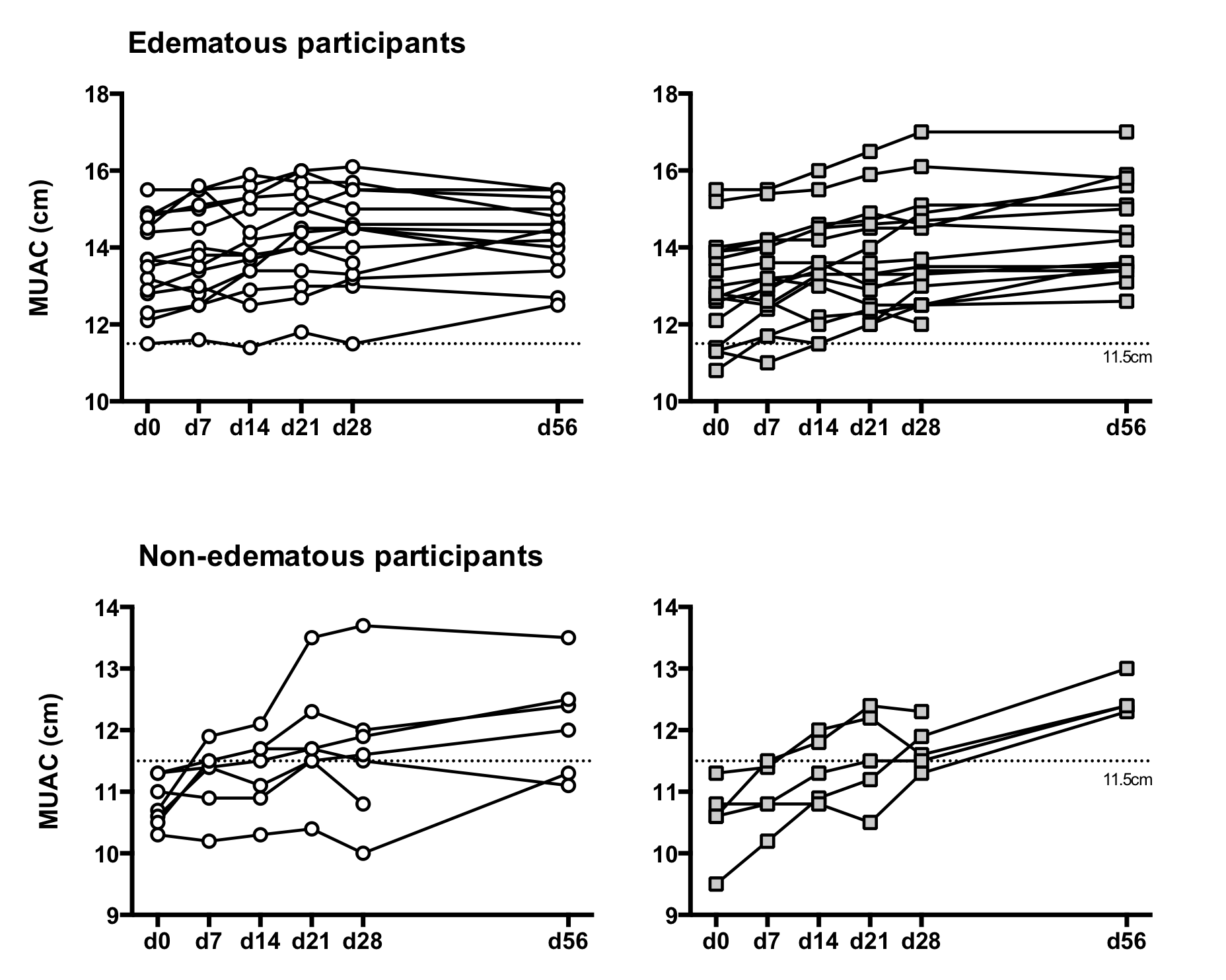
**

**Supplementary Figure 2:**


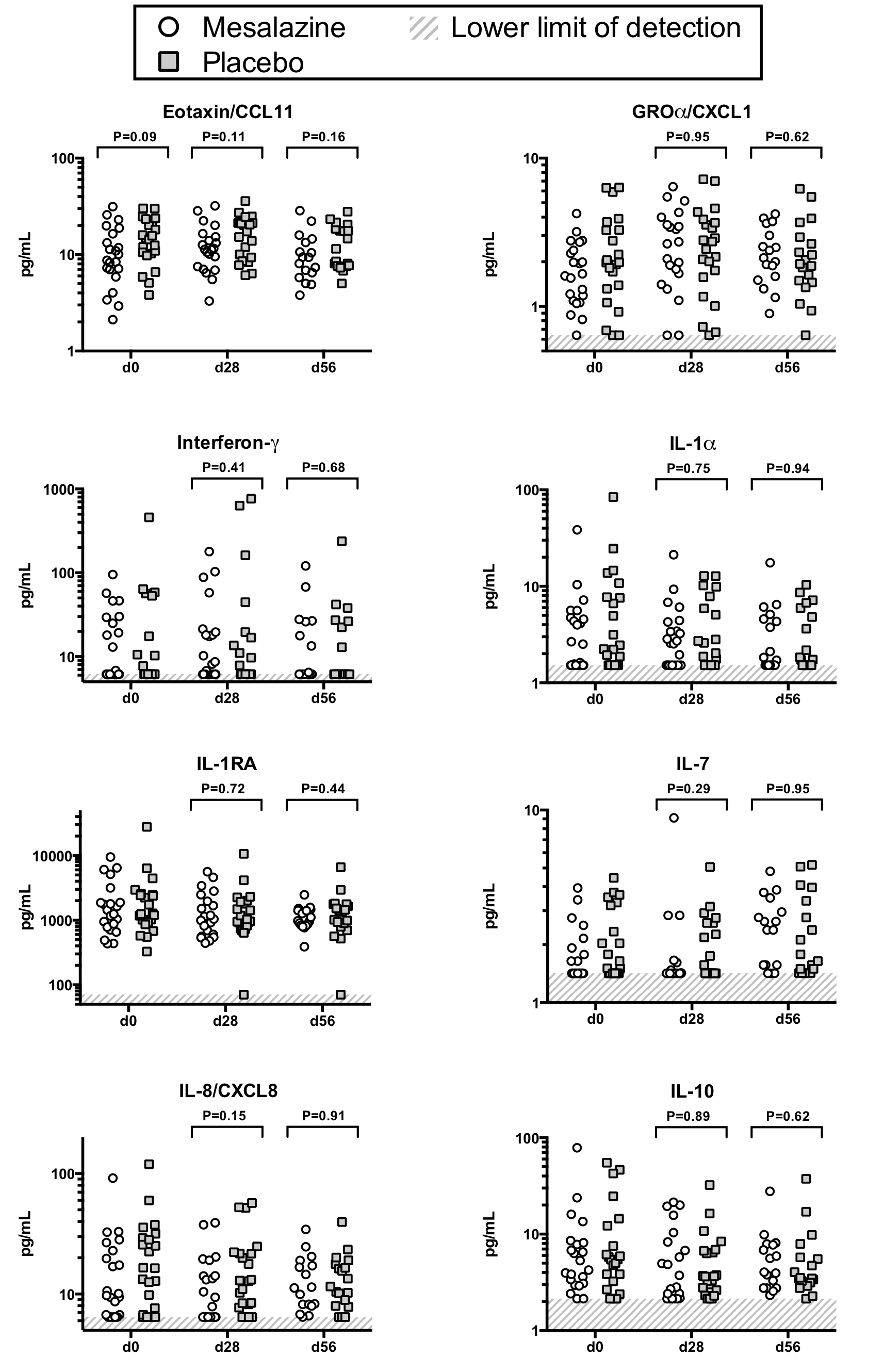


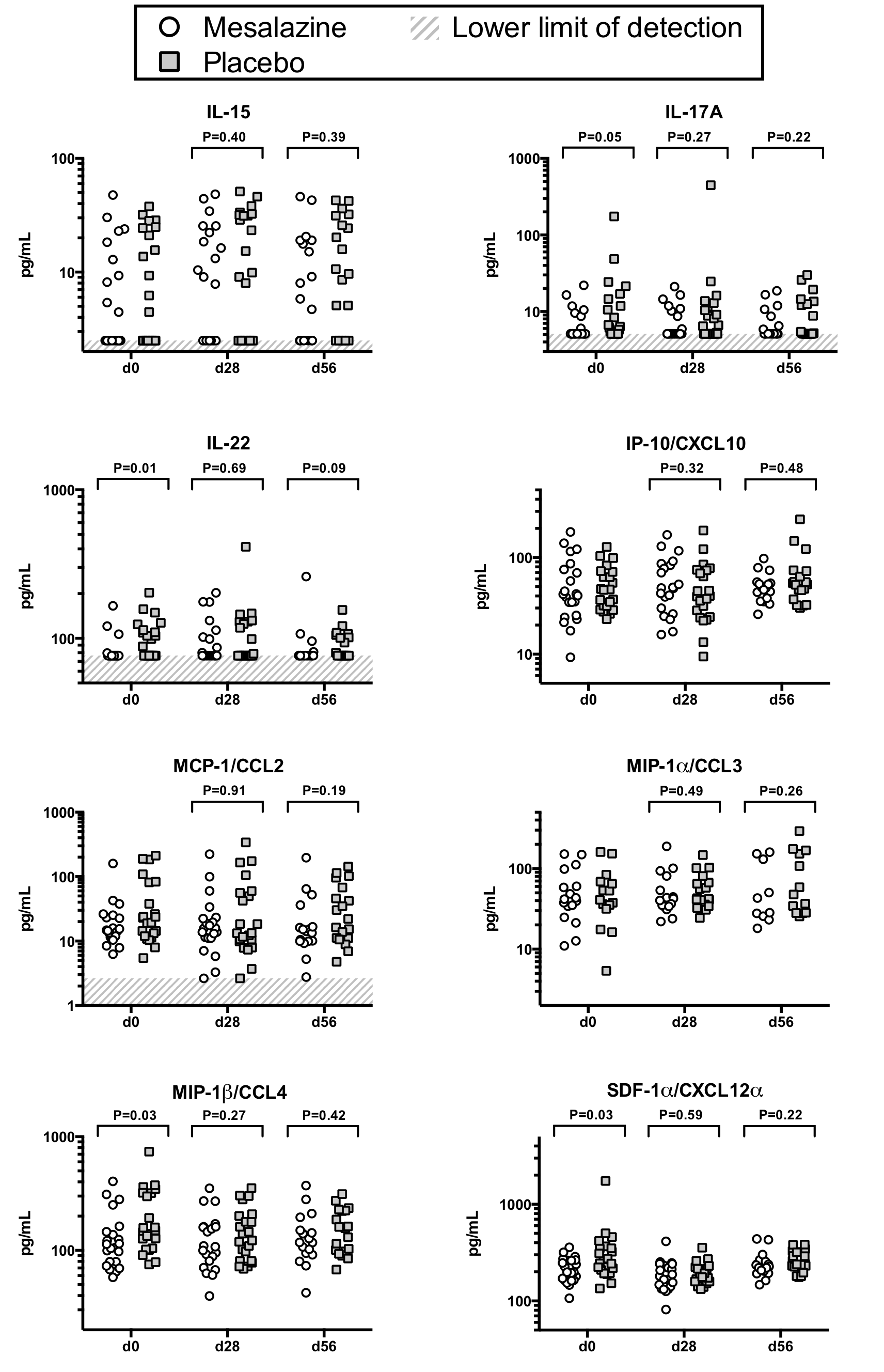

Supplement: Additional file 1: — Swahili version of Bristol Stool Form Scale. Figure S1. Individual mid-upper arm circumference (MUAC) trajectories for participants in the mesalazine arm (left) or placebo arm (right), grouped according to whether they were edematous (top) or non-edematous (bottom) at baseline assessment. Figure S2. Plasma cytokine concentrations between the arms. Differences between arms at baseline are highlighted when P <0.1. [file 12916_2014_133_MOESM1_ESM.doc]
